# Effective population size of *Anopheles funestus *chromosomal forms in Burkina Faso

**DOI:** 10.1186/1475-2875-5-115

**Published:** 2006-11-24

**Authors:** Andrew P Michel, Olga Grushko, Wamdaogo M Guelbeogo, N'Fale Sagnon, Carlo Costantini, Nora J Besansky

**Affiliations:** 1 Center for Global Health and Infectious Diseases, Department of Biological Sciences, University of Notre Dame, Notre Dame, IN 46628-0369, USA; 2 Centre National de Recherche et Formation sur le Paludisme, 01 BP 2208 Ouagadougou 01, Burkina Faso; 3 Institut de Recherche pour le Développement, Ouagadougou 01, Burkina Faso

## Abstract

**Background:**

As *Anopheles funestus *is one of the principal Afro-tropical malaria vectors, a more complete understanding of its population structure is desirable. In West and Central Africa, *An. funestus *population structure is complicated by the coexistence of two assortatively mating chromosomal forms. Effective population size (*N*_*e*_) is a key parameter in understanding patterns and levels of intraspecific variation, as it reflects the role of genetic drift. Here, *N*_*e *_was estimated from both chromosomal forms, Kiribina and Folonzo, in Burkina Faso.

**Methods:**

Short-term *N*_*e *_was estimated by evaluating variation at 16 microsatellite loci across temporal samples collected annually from 2000–2002. Estimates were based on standardized variance in allele frequencies or a maximum likelihood method. Long-term *N*_*e *_was estimated from genetic diversity estimates using mtDNA sequences and microsatellites.

**Results:**

For both forms, short-term and long-term *N*_*e *_estimates were on the order of 10^3 ^and 10^5^, respectively. Long-term *N*_*e *_estimates were larger when based on loci from chromosome 3R (both inside and outside of inversions) than loci outside of this arm.

**Conclusion:**

*N*_*e *_values indicate that *An. funestus *is not subject to seasonal bottlenecks. Though not statistically different because of large and overlapping confidence intervals, short-term *N*_*e *_estimates were consistently smaller for Kiribina than Folonzo, possibly due to exploitation of different breeding sites: permanent for Folonzo and intermittent for Kiribina. The higher long-term *N*_*e *_estimates on 3R, the arm carrying the two inversions mainly responsible for defining the chromosomal forms, give natural selection broader scope and merit further study.

## Background

The efficient application of malaria control methods that target the mosquito vector depends upon knowledge of its population genetic structure. This information can improve current insecticide-based strategies and aid in the management of insecticide resistance, but it is also essential to future genetic control or modification strategies that aim to reduce, eliminate or replace vector populations with non-vectors. Unfortunately, present understanding of the population structure of any malaria vector is insufficient to underpin a genetic control programme, and nowhere is this shortfall more critical than in sub-Saharan Africa where three widespread species (*Anopheles gambiae*, *Anopheles arabiensis *and *Anopheles funestus*) are responsible for transmitting most of the 1–3 million fatal cases each year [1].

*An. funestus*, one of the most anthropophilic vectors known, exploits permanent or semi-permanent breeding sites such as marshes or rice fields. Its population density peaks in the dry season, extending malaria transmission by relay after *An. gambiae *and *An. arabiensis *populations have declined [2]. A highly polymorphic species, its population structure appears quite shallow across continental Africa. Evidence from microsatellite and mtDNA markers suggests a division between populations on either side of the Great Rift Valley complex, but little differentiation among populations within these regions, even between locations spanning several thousand kilometers [3]. However, in Burkina Faso, West Africa, analysis of polymorphic chromosomal inversions has revealed cryptic complexities in population structure. A temporally and spatially stable pattern of inversion polymorphism in sympatric and synchronous samples of *An. funestus *is inconsistent with random mating, suggesting the presence of two assortatively mating chromosomal forms designated Folonzo and Kiribina [4,5]. The relative abundance of Kiribina near rice cultivation and Folonzo near marshy areas suggests some partitioning of larval habitats, and the latter form is more likely to rest indoors, feed on humans, and be infected with malaria parasites [4]. At the molecular level, differentiation between forms is slight and is accounted for mainly by markers mapping to the chromosome arm (3R) bearing the principal inversions whose frequencies define the forms [6,7]. The working hypothesis is that Folonzo and Kiribina forms of *An. funestus *are incipient species whose distinctions are linked, at least in part, to chromosomal inversions.

Effective population size (*N*_e_) is a central parameter in the description of population structure, though notoriously difficult to estimate with precision [8]. *N*_e _is inversely related to genetic drift, the rate of fluctuation in allele frequencies caused by random sampling [9], and can be defined as "the size of an ideal population that exhibits the same rate of drift as the actual population it characterizes" [10]. In an ideal population, one which is randomly mating, constant in size and uniform in reproductive potential, *N*_e _is equal to N, the census population size. In actual populations, *N*_e _is usually less than N, because of large variance in reproductive success among individuals. This is especially true where population size fluctuates seasonally, as is the case for the primary malaria vectors of Africa, because *N*_e _approximates the harmonic mean of single-generation sizes and, therefore, more closely reflects the lowest values. *N*_e _has been estimated for *An. gambiae *and *An. arabiensis *by a number of authors in different parts of Africa using indirect genetic methods and direct mark-release-recapture [11-16]; values were typically at least 10^3^, inconsistent with seasonal bottlenecks. Because no estimates were available for *An. funestus*, *N*_e _was estimated for Folonzo and Kiribina forms in Burkina Faso using temporal variation at 16 microsatellite loci across 3 consecutive years, and sequence variation in an 834 bp region of the mitochondrial DNA *ND5 *gene.

## Materials and methods

### Mosquitoes

Indoor-resting insecticide spray-sheet collections were performed in December of three consecutive years (2000, 2001, and 2002) in Koubri-Kuiti, Burkina Faso (12°11'N; 1°23'W), as previously described [7]. Karyotyping and chromosomal form identification followed methods of Guelbeogo *et al *.[5].

### Microsatellites and mtDNA

Genomic DNA was extracted from single mosquito carcasses [7]. Prior to analysis by PCR, genomic DNA was diluted 1:10 in H_2_O (~5 ng/ul). Morphological identification of *An. funestus *was verified on each specimen by a modified rDNA-based PCR assay [7].

For 2001, microsatellite data of Michel *et al*. [7] were taken from 50 randomly chosen specimens of both chromosomal forms. For 2000 and 2002, the same 16 physically mapped microsatellites were PCR amplified from ~50 specimens of each form (exact sample size given in [Additional file 1]). Products were diluted, pool-plexed (two groups of eight loci each), genotyped on a Beckman-Coulter CEQ8000, and sized with software provided (see [7] for detailed methods). *Fstat*2.9.3.2 [17] was used to estimate allelic richness (*R*_s_), deviations from Hardy-Weinberg equilibrium (inbreeding coefficient, *F*_IS_) and linkage disequilibrium. The impact of suspected null alleles was explored by using MICRO-CHECKER [18] to adjust allele and genotype frequencies based on the Brookfield 2 estimate [19], followed by repeated analyses on the null allele-adjusted data set, as described previously [7]. No significant changes in outcome were observed. Microsatellite differentiation (F_ST_) between Folonzo and Kiribina samples from each year was computed with Microsatellite Analyzer [20].

Mitochondrial sequences (834 bp of the *ND5 *gene) were taken from Michel et al. [6], based on 90 Folonzo and 96 Kiribina samples collected from La and Pehele, Burkina Faso in 2002 [GenBank, Kiribina: DQ126772–DQ126867; Folonzo: DQ127048–DQ127137]. There was no significant mtDNA or microsatellite differentiation between these locales for samples of the same chromosomal form [6].

### Estimates of short-term *N*_e_

Short-term *N*_e _was estimated using temporal changes in microsatellite allele frequencies. Under the assumption of no mutation, selection or migration, changes in allele frequency are the result of genetic drift, whose strength is inversely related to population size. Both a moment estimator (based on the standardized variance in allele frequency change, *F *[21]) and a probability method (the maximum likelihood method of [22]) were used to calculateshort-term *N*_e _from temporal samples. Although the probabilistic approach has been shown to have higher accuracy and precision [8], the moment method of Waples [21] (hereafter, *F*-statistic method) was the approach adopted for estimates in other vectors; including it facilitated comparison of *N*_e _between vector species. The *F*-statistic method was implemented using the software programme NeEstimator [23]. The maximum likelihood method (hereafter, ML method) was implemented using the programme MLNE 2.0 [24]. To reduce computational burden, maximum population size was set initially at 15,000. Subsequently, analyses were repeated using a maximum size of 25,000. In both cases, the upper bound of the 95% confidence interval (CI) always reached the maximum limit for Folonzo (and did so for one sampling period for Kiribina). Thus, the upper bound CI was not determined in these cases. Following other authors [12-15], *N*_e _was evaluated under the conservative assumption of 12 generations per year, but the effect of fewer (10) or more (20) generations per year was also explored.

### Estimates of long-term *N*_e_

Long-term *N*_e _was estimated from current genetic variation (θ = 4*N*_e_μ for autosomal loci, where μ is mutation rate), under the assumption of mutation-drift equilibrium (MDE). For microsatellites, current genetic variation was represented by Xu and Fu's [25] estimator, θ_F_, based on sample homozygosity under the single-step stepwise mutation model. The average mutation rate for dinucleotide microsatellite repeats is unknown for *An. funestus*, as for *An. gambiae*. In the latter species, an upper-bound estimate of 10^-4 ^mutations per locus per generation based on the data of Zheng et al. [26,27] has been used [12], but this value is likely an overestimate. Accordingly, a slower mutation rate of 9.3 × 10^-6 ^mutations per microsatellite locus per generation was adopted, based on the estimates derived from dinucleotide microsatellites in *Drosophila melanogaster *[28]. Mitochondrial values of θ were estimated from mean pairwise sequence differences per site (π [29]) and from the number of segregating sites among sequences (*S *[29]) as calculated in *DNASP *v 4.10.1 [30]. Assuming a mutation rate of 5.7 × 10^-8 ^per base per gamete [31], long-term *N*_e _was estimated using the equation θ = *N*_e_μ.

## Results and discussion

Sample sizes and summary polymorphism statistics are presented in [Additional file 1]. Allelic richness and heterozygosity were relatively high across years and chromosomal forms (mean *R*_s _per locus ranged from 4.4 to 21.0; mean *H*_o _per locus ranged from 0.41 to 0.81). All loci were found to be in linkage equilibrium. Significant Hardy-Weinberg deficits occurred in 12 of 96 possible tests, but were clustered at 3 loci (AFND12, FunD, and AFUB12) suspected of harboring null alleles [6,7].

For all three sampling years there was slight but significant differentiation between the chromosomal forms, with frequency differences between loci on chromosome 3R (both inside and outside of inversions) accounting for much but not all of the differentiation (Table 1).

**Table 1:** Microsatellite F_ST _values between *An. funestus *chromosomal forms at each of three sampling years

	Year 2000	Year 2001	Year 2002
All loci (16)	0.007***	0.006***	0.011***
Non-3R loci (11)	-0.013	0.005*	0.007**
3R Loci (5)	0.011***	0.007***	0.018***

*P < 0.05; **P < 0.01; ***P < 0.001

### Short-term *N*_e_

Table 2 presents estimates of short-term *N*_e _across sampling intervals of one year (2000–2001, 2001–2002) and two years (2000–2002) for both chromosomal forms. The number of generations per year is uncertain, but 12 are plausible and the actual number should fall within the extreme values employed of 10 to 20. Between these values, all *N*_e _estimates were on the order of 10^3^, indicating the absence of seasonal bottlenecks. This is consistent with results from *An. gambiae *and *An. arabiensis*, even under severe climatic conditions, on islands, and following insecticide spray campaigns (*e.g*., [13,14]).

**Table 2:** Short-term *N*_e _based on temporal changes in microsatellite allele frequency

	10 generations per year		12 generations per year		20 generations per year	
Sampling Interval	Folonzo *N*_e_-F^a ^(95% CI) *N*_e_-ML^b ^(95% CI)	Kiribina *N*_e_-F (95% CI) *N*_e_-ML (95% CI)	Folonzo *N*_e_-F^a ^(95% CI) *N*_e_-ML^b ^(95% CI)	Kiribina *N*_e_-F (95% CI) *N*_e_-ML (95% CI)	Folonzo *N*_e_-F^a ^(95% CI) *N*_e_-ML^b ^(95% CI)	Kiribina *N*_e_-F (95% CI) *N*_e_-ML (95% CI)

2000–2001	2,083 (665-∞)	811 (397-3,323)	2,500 (798-∞)	973 (476-3,987)	4,167 (1,330-∞)	1,622 (1,330-∞)
	2,736 (807-ND^c^)	1,081 (507-11,836)	3,248 (967-ND^c^)	1,288 (605-13,747)	5,300 (1,616-ND^c^)	2,120 (987-ND^c^)
2001–2002	2,166 (649-∞)	744 (357-3,324)	2,599 (779-∞)	893 (428-3,989)	4,332 (1,299-∞)	1,488 (714-6,648)
	1,876 (685-ND^c^)	588 (324-1,338)	2,227 (813-ND^c^)	597 (347-1,391)	3,637 (1,309-ND^c^)	982 (571-2,282)
2000–2002	7,363 (1,635-∞)	941 (543–1,990)	8,835 (1,963-∞)	1,129 (651–2,389)	14,726 (3,271-∞)	1,882 (1,086–3,981)
	3,404 (1,430-ND^c^)	865 (571–1,512)	4,064 (1707-ND^c^)	1,034 (682–1,807)	3,404 (1,430-ND^c^)	1,710 (1,127–2,988)

^a^*N*_e _estimated based on the standardized variance in allele frequency change (F) [21]

^b^*N*_e _estimated using the maximum likelihood (ML) approach implemented in MLNE 2.0 [24]

^c^ND, not determined. See methods.

Regardless of the estimation method, *N*_e _values for the Kiribina form at each time point were at least 2–3 times smaller than those for the Folonzo form, though the 95% CIs overlapped. The upper bound of the 95% CI for Folonzo was invariably infinity under the *F*-statistic method and was not determined under the ML method because it exceeded the maximum value imposed (25,000) during implementation of MLNE 2.0. With one exception (under the unrealistic case of 20 generations per year), the upper bound of the 95% CI for Kiribina was always defined, and ranged from ~1,300–14,000. Ecological data concerning the differences between these chromosomal forms is very incomplete, owing to the lack of molecular markers and the consequent necessity of determining karyotype from semi-gravid females by laborious cytogenetic methods. Preliminary indications are that Kiribina may prefer anthropogenic breeding sites such as rice fields, whereas Folonzo predominates in association with more natural and permanent habitats such as marshes and swamps. If confirmed by follow-up studies, the different breeding habitats may help explain differences in *N*_e_, because rice fields do not produce anophelines continuously. Rice fields are flooded in June/July and harvested in October/November, with a second crop sometimes grown between March and June. Even during these intervals, the ability of *An. funestus *to exploit rice fields depends upon the stage of rice growth [32].

Estimates of *N*_e _produced by the *F*-statistic and ML methods were very similar, except for the two-year sampling interval for the Folonzo form, where *N*_e _values estimated from *F *were several-fold larger than those from the ML method and were inconsistent with both of the corresponding one-year sampling intervals. Unlike the ML method, the *F*-statistic method is known to have an upward bias if rare alleles are present, and it is possible that the longer sampling interval resulted in more rare alleles that led to an overestimate of *N*_e _[22]. Because of the inconsistent performance of the *F*-statistic method and the greater precision of the ML method [8], ML estimates of *N*_e _should be given greater weight. However, it should be noted that both methods assume the absence of mutation, selection and migration. While the first assumption is not unreasonable over short sampling intervals, and direct selection on microsatellite markers seems unlikely, the assumption of an isolated population without immigration may not be [24]. In the short-term, ignoring immigration, if it actually exists, leads to underestimates of *N*_e _[8]. Both methods also assume constant population size and discrete generations, which are not realistic for *An. funestus*. Fluctuating population size with overlapping generations can lead to overestimates of *N*_e _[33].

### Long-term *N*_e_

Unlike short-term *N*_e _that reflects recent effects on genetic variation in a focal population, long-term *N*_e _reflects evolutionary forces and demographic processes over a much greater geographic and historical frame (on the order of *N*_e _generations; [8]), thus it approaches the effective size of the entire species. Long-term *N*_e _was estimated from genetic diversity based on microsatellites and mtDNA sequences from samples collected in 2002 (Table 3). Microsatellite data from other years gave very similar estimates and, therefore, are not shown. As expected, long-term estimates are 2 orders of magnitude larger than the short-term *N*_e _values (10^5 ^versus 10^3^). Moreover, the estimates from microsatellites and mtDNA are roughly in accord. Departing from recent convention for estimates of *N*_e _in other Afro-tropical vector species, 9.3 × 10^-6 ^was assumed as the mutation rate for microsatellites, an order of magnitude slower than the upper-bound estimate for *An. gambiae *[12] but consistent with measurements from *D. melanogaster *[28]. The mtDNA mutation rate was assumed to be 5.7 × 10^-8^. The uncertainties in these mutation rates as applied to *An. funestus *mean that the long-term *N*_e _values lack precision. It also should be noted that because of recent population expansion, *An. funestus *populations west of the Rift Valley (including those from Burkina Faso) violate the assumption of MDE under which long-term *N*_e _is derived [3,6]; the departure from MDE is suggested by divergent estimates of θ from *S *or π seen in Table 3. The degree to which the long-term estimates of *N*_e _are biased by population expansion is not known. However, long-term *N*_e _estimates from Burkina Faso can be compared to long-term estimates from countries east of the Rift Valley where the evidence does not support a population expansion [3]. Using values of gene diversity derived from the data of Michel et al [3], who employed 10 of the 16 microsatellite loci used in the present study (only two on 3R), long-term *N*_e _values from Malawi or Tanzania samples were smaller by about one-third (59,340 and 49,000) relative to values from Burkina Faso based on the same 10 loci (Kiribina: 160,940; Folonzo: 169,700).

**Table 3:** Long-term *N*_e _based on genetic diversity estimates (θ) from 2002

	**Folonzo**		**Kiribina**	
**Locus**	θ	N_e_	θ	N_e_

Microsatellites				
All loci (16)	0.79^a^	250,590	0.77^a^	202,030
Non-3R loci (11)	0.76^a^	174,860	0.75^a^	174,420
3R loci (5)	0.85^a^	412,880	0.81^a^	262,780
				
mtDNA	0.00534^b^	93,684	0.00563^b^	98,771
	0.01939^c^	340,175	0.01774^c^	311,228

^a^θ_F _[25]

^b^Mean pairwise nucleotide difference per base, π [29]

^c^Segregating sites per base, *S *[29]

Comparison between Folonzo and Kiribina *N*_e _values is not hindered by uncertainty in mutation rate, as this rate should be the same for both forms at a given set of loci. The *N*_e _estimates from mtDNA are essentially identical between the forms. Across all 16 microsatellite loci, it appears that *N*_e _for Folonzo is slightly larger than that for Kiribina. However, partitioning the loci into two groups – those residing on chromosome 3R or those that map elsewhere in the genome – reveals that the difference between forms can be explained by loci on 3R, the arm that carries the two main chromosomal inversions involved in distinguishing the forms [4,5]. There is higher diversity on this arm in Folonzo than in Kiribina. This is not altogether surprising, as the deterministic algorithm which defines these forms allows more polymorphism in Folonzo with respect to alternative arrangements on 3R. However, this is not the entire story. The relative level of genetic diversity at the 5 loci on 3R versus the 11 loci outside 3R is significantly higher in *both *forms (Mann-Whitney test: Folonzo U = 43.5, *P *< 0.03; Kiribina U = 49.5, *P *< 0.01). The reason(s) for this pattern are not clear from the present data, but higher genetic diversity provides more scope to natural selection and further investigation of chromosome 3R seems warranted.

## Conclusion

The present estimates of effective population size for *An. funestus*, though approximate, preclude strong seasonal bottlenecks. Based on microsatellite variation between temporal samples of *An. funestus *in Burkina Faso, short-term estimates of *N*_e _were on the order of 10^3 ^and were consistently smaller for the Kiribina than the Folonzo chromosomal form, possibly related to the preference of Kiribina for breeding in rice fields that are not in continuous production. Long-term estimates of *N*_e _were consistent between classes of marker (microsatellites or mtDNA), and were two orders of magnitude larger than short-term estimates. Although long-term *N*_e _did not differ between chromosomal forms, in each case there was significantly higher genetic diversity on the chromosome arm (3R) that carries the principal inversions defining the two taxa, a finding that merits further study.

As *N*_e _reflects the strength of genetic drift, it is a central parameter in descriptions of population genetic structure. The *N*_e _estimates derived here provide some insight into the complex population structure of *An. funestus *in Burkina Faso where the two chromosomal forms coexist. However, the geographic extent and nature of these forms has not been well characterized outside of Burkina Faso. In general, *An. funestus *is a heterogeneous species that occupies diverse habitats across Africa, where its population structure and history may differ. A more complete understanding of the forces that structure genetic variation in *An. funestus *will depend upon additional studies in other parts of its range.

## Authors' contributions

APM participated in field collections, conducted the microsatellite genotyping, performed the data analysis, and drafted the manuscript. OG performed karyotype analysis and mtDNA sequencing and analysis. WG participated in collection efforts and performed karyotype analysis. N'FS, CC and NJB participated in the design and coordination of the study. NJB participated in drafting the manuscript. All authors read and approved the final manuscript.

## Supplementary Material

Additional file 1 Microsatellite variation at 16 loci from two chromosomal forms of *An. funestus *during three consecutive sampling years in Burkina Faso, West Africa. The data provided in this table represent polymorphism statistics estimated at each of 16 microsatellite loci from population samples of *An. funestus *collected in Burkina Faso. Click here for file

## References

[B1] Coluzzi M (1999). The clay feet of the malaria giant and its African roots: hypotheses and inferences about origin, spread and control of Plasmodium falciparum.. Parassitologia.

[B2] Gillies MT, De Meillon B (1968). The Anophelinae of Africa South of the Sahara.

[B3] Michel AP, Ingrasci MJ, Schemerhorn BJ, Kern M, Le Goff G, Coetzee M, Elissa N, Fontenille D, Vulule J, Lehmann T, Sagnon N, Costantini C, Besansky NJ (2005). Rangewide population genetic structure of the African malaria vector Anopheles funestus. Mol Ecol.

[B4] Costantini C, Sagnon NF, Ilboudo-Sanogo E, Coluzzi M, Boccolini D (1999). Chromosomal and bionomic heterogeneities suggest incipient speciation in Anopheles funestus from Burkina Faso. Parassitologia.

[B5] Guelbeogo WM, Grushko O, Boccolini D, Ouedraogo PA, Besansky NJ, Sagnon NF, Costantini C (2004). Chromosomal evidence of incipient speciation in the Afrotropical malaria mosquito Anopheles funestus. Med Vet Entomol.

[B6] Michel AP, Grushko O, Guelbeogo WM, Lobo NF, Sagnon N, Costantini C, Besansky NJ (2006). Divergence with gene flow in Anopheles funestus from the Sudan Savanna of Burkina Faso, West Africa. Genetics.

[B7] Michel AP, Guelbeogo WM, Grushko O, Schemerhorn BJ, Kern M, Willard MB, Sagnon N, Costantini C, Besansky NJ (2005). Molecular differentiation between chromosomally defined incipient species of Anopheles funestus. Insect Mol Biol.

[B8] Wang J (2005). Estimation of effective population sizes from data on genetic markers. Philos Trans R Soc Lond B Biol Sci.

[B9] Charlesworth B (2002). Effective population size. Curr Biol.

[B10] Taylor CE, Manoukis NC, Takken W, Scott TW (2003). Effective population size in relation to genetic modification of Anopheles gambiae sensu stricto. Ecological Aspects for Application of Genetically Modified Mosquitoes.

[B11] Costantini C, Li SG, Della Torre A, Sagnon N, Coluzzi M, Taylor CE (1996). Density, survival and dispersal of Anopheles gambiae complex mosquitoes in a west African Sudan savanna village. Med Vet Entomol.

[B12] Lehmann T, Hawley WA, Grebert H, Collins FH (1998). The effective population size of Anopheles gambiae in Kenya: implications for population structure. Mol Biol Evol.

[B13] Pinto J, Donnelly MJ, Sousa CA, Malta-Vacas J, Gil V, Ferreira C, Petrarca V, do Rosario VE, Charlwood JD (2003). An island within an island: genetic differentiation of Anopheles gambiae in Sao Tome, West Africa, and its relevance to malaria vector control. Heredity.

[B14] Simard F, Lehmann T, Lemasson JJ, Diatta M, Fontenille D (2000). Persistence of Anopheles arabiensis during the severe dry season conditions in Senegal: an indirect approach using microsatellite loci. Insect Mol Biol.

[B15] Taylor CE, Toure YT, Coluzzi M, Petrarca V (1993). Effective population size and persistence of Anopheles arabiensis during the dry season in west Africa. Med Vet Entomol.

[B16] Toure YT, Dolo G, Petrarca V, Traore SF, Bouare M, Dao A, Carnahan J, Taylor CE (1998). Mark-release-recapture experiments with Anopheles gambiae s.l. in Banambani Village, Mali, to determine population size and structure. Med Vet Entomol.

[B17] Goudet J (2001). FSTAT, a program to estimate and test gene diversities and fixation indices (version 2.9.3).. http://www2.unil.ch/popgen/softwares/fstat.htm.

[B18] van Oosterhout C, Hutchinson WF, Wills DPM, Shipley PF (2004). Micro-Checker: Software for identifying and correcting genotyping errors in microsatellite data.. Mol Ecol Notes.

[B19] Brookfield JF (1996). A simple new method for estimating null allele frequency from heterozygote deficiency. Mol Ecol.

[B20] Dieringer D, Schlotterer C (2003). Microsatellite analyzer (MSA): a platform independent analysis tool for large microsatellite data sets.. Mol Ecol Notes.

[B21] Waples RS (1989). A generalized approach for estimating effective population size from temporal changes in allele frequency. Genetics.

[B22] Wang J (2001). A pseudo-likelihood method for estimating effective population size from temporally spaced samples. Genet Res.

[B23] Peel D, Ovenden JR, Peel SL (2004). NeEstimator 1.3: software for estimating effective population size.

[B24] Wang J, Whitlock MC (2003). Estimating effective population size and migration rates from genetic samples over space and time. Genetics.

[B25] Xu H, Fu YX (2004). Estimating effective population size or mutation rate with microsatellites. Genetics.

[B26] Zheng L, Benedict MQ, Cornel AJ, Collins FH, Kafatos FC (1996). An integrated genetic map of the African human malaria vector mosquito, Anopheles gambiae. Genetics.

[B27] Zheng L, Cornel AJ, Wang R, Erfle H, Voss H, Ansorge W, Kafatos FC, Collins FH (1997). Quantitative trait loci for refractoriness of Anopheles gambiae to Plasmodium cynomolgi B. Science.

[B28] Schug MD, Hutter CM, Wetterstrand KA, Gaudette MS, Mackay TF, Aquadro CF (1998). The mutation rates of di-, tri- and tetranucleotide repeats in Drosophila melanogaster. Mol Biol Evol.

[B29] Nei M (1987). Molecular Evolutionary Genetics.

[B30] Rozas J, Sanchez-DelBarrio JC, Messeguer X, Rozas R (2003). DnaSP, DNA polymorphism analyses by the coalescent and other methods. Bioinformatics.

[B31] Tamura K (1992). The rate and pattern of nucleotide substitution in Drosophila mitochondrial DNA. Mol Biol Evol.

[B32] Diuk-Wasser MA, Toure MB, Dolo G, Bagayoko M, Sogoba N, Traore SF, Manoukis N, Taylor CE (2005). Vector abundance and malaria transmission in rice-growing villages in Mali. Am J Trop Med Hyg.

[B33] Waples RS (2002). Effective size of fluctuating salmon populations. Genetics.

