# Supplementary material for: Effective population size of Anopheles funestus chromosomal forms in Burkina Faso
Source: Malar J. 2006 Nov 24;5:115. doi: 10.1186/1475-2875-5-115 (PMC1676016; doi:10.1186/1475-2875-5-115)
Supplement: Additional file 1 — Microsatellite variation at 16 loci from two chromosomal forms of An. funestus during three consecutive sampling years in Burkina Faso, West Africa. The data provided in this table represent polymorphism statistics estimated at each of 16 microsatellite loci from population samples of An. funestus collected in Burkina Faso. [file 1475-2875-5-115-S1.doc]

Additional file 1. Microsatellite variation at 16 loci from two chromosomal forms of *An. funestus* during three consecutive sampling years in Burkina Faso, West Africa.

|  | **Folonzo** | | |  | **Kiribina** | | |
| --- | --- | --- | --- | --- | --- | --- | --- |
| **Locus** | **2000**  **(57)** | **2001**  **(50)** | **2002**  **(53)** |  | **2000**  **(56)** | **2001**  **(50)** | **2002**  **(47)** |
| **AF12 (X)** |  |  |  |  |  |  |  |
| Rs | 9.6 | 10.8 | 9.8 |  | 9.4 | 10.9 | 9.0 |
| Ho | 0.72 | 0.64 | 0.65 |  | 0.54 | 0.67 | 0.71 |
| Fis | 0.15 | **0.24** | 0.23 |  | **0.36** | **0.21** | 0.15 |
| **FUNQ (X)** |  |  |  |  |  |  |  |
| Rs | 5.8 | 6.0 | 6.7 |  | 5.9 | 7.9 | 6.0 |
| Ho | 0.56 | 0.60 | 0.66 |  | 0.65 | 0.51 | 0.71 |
| Fis | 0.24 | 0.15 | 0.10 |  | 0.08 | 0.35 | 0.04 |
| **AF40 (2R)** |  |  |  |  |  |  |  |
| Rs | 7.6 | 6.0 | 7.7 |  | 5.6 | 8.0 | 6.9 |
| Ho | 0.70 | 0.72 | 0.81 |  | 0.66 | 0.68 | 0.73 |
| Fis | 0.08 | 0.03 | -0.10 |  | 0.02 | 0.10 | -0.01 |
| **AFND32 (2R)** |  |  |  |  |  |  |  |
| Rs | 11.3 | 8.9 | 9.5 |  | 8.8 | 10.8 | 8.0 |
| Ho | 0.84 | 0.62 | 0.64 |  | 0.65 | 0.65 | 0.59 |
| Fis | -0.01 | 0.23 | 0.19 |  | 0.17 | 0.21 | 0.27 |
| **FUNO (2R)** |  |  |  |  |  |  |  |
| Rs | 9.5 | 10.8 | 9.5 |  | 10.8 | 10.8 | 9.0 |
| Ho | 0.74 | 0.70 | 0.77 |  | 0.77 | 0.63 | 0.66 |
| Fis | 0 | 0.08 | -0.07 |  | 0.05 | 0.16 | 0.14 |
| **AFUB10 (2L)** |  |  |  |  |  |  |  |
| Rs | 7.9 | 7.9 | 10.5 |  | 10.6 | 8.0 | 8.0 |
| Ho | 0.70 | 0.74 | 0.81 |  | 0.73 | 0.69 | 0.67 |
| Fis | 0.11 | 0.10 | 0.01 |  | 0.10 | 0.16 | 0.18 |
| **FUNL (2L)** |  |  |  |  |  |  |  |
| Rs | 14.6 | 16.4 | 13.4 |  | 12.7 | 12.9 | 14.8 |
| Ho | 0.67 | 0.66 | 0.81 |  | 0.68 | 0.67 | 0.65 |
| Fis | 0.17 | 0.18 | 0.04 |  | 0.20 | 0.18 | 0.24 |
| **AFND23 (2L)** |  |  |  |  |  |  |  |
| Rs | 9.6 | 11.8 | 10.6 |  | 8.0 | 9.0 | 8.0 |
| Ho | 0.81 | 0.74 | 0.66 |  | 0.71 | 0.79 | 0.81 |
| Fis | 0.06 | 0.12 | 0.21 |  | 0.14 | 0.05 | 0.04 |
| **UB11 (2L)** |  |  |  |  |  |  |  |
| Rs | 9.5 | 8.8 | 8.7 |  | 6.8 | 8.9 | 8.0 |
| Ho | 0.63 | 0.56 | 0.68 |  | 0.73 | 0.60 | 0.63 |
| Fis | 0.08 | 0.17 | 0.04 |  | -0.03 | 0.16 | 0.15 |
| **AFND20 (3R)** |  |  |  |  |  |  |  |
| Rs | 11.2 | 9.8 | 10.6 |  | 11.2 | 8.9 | 6.9 |
| Ho | 0.79 | 0.64 | 0.85 |  | 0.79 | 0.77 | 0.85 |
| Fis | 0.06 | 0.20 | 0 |  | 0 | 0.05 | -0.15 |
| **AF7 (3R)** |  |  |  |  |  |  |  |
| Rs | 8.8 | 8.8 | 6.9 |  | 8.6 | 7.9 | 8.0 |
| Ho | 0.84 | 0.70 | 0.69 |  | 0.82 | 0.59 | 0.79 |
| Fis | 0.03 | 0.13 | 0.13 |  | 0.01 | **0.28** | 0.04 |
| **AFND19 (3R)** |  |  |  |  |  |  |  |
| Rs | 9.8 | 10.9 | 9.0 |  | 11.4 | 9.9 | 9.0 |
| Ho | 0.75 | 0.78 | 0.87 |  | 0.71 | 0.65 | 0.70 |
| Fis | 0.07 | 0.04 | -0.07 |  | 0.08 | 0.06 | 0.10 |
| **FUNG (3R)** |  |  |  |  |  |  |  |
| Rs | 15.1 | 16.7 | 13.6 |  | 11.25 | 11.9 | 10.9 |
| Ho | 0.86 | 0.80 | 0.87 |  | 0.79 | 0.83 | 0.71 |
| Fis | 0.02 | 0.11 | 0.03 |  | 0.06 | 0.03 | 0.13 |
| **FUND (3R)** |  |  |  |  |  |  |  |
| Rs | 23.7 | 23.5 | 22.0 |  | 20.3 | 18.8 | 17.9 |
| Ho | 0.55 | 0.72 | 0.64 |  | 0.68 | 0.82 | 0.68 |
| Fis | **0.38** | **0.21** | **0.30** |  | **0.23** | 0.09 | **0.24** |
| **AFUB12 (3L)** |  |  |  |  |  |  |  |
| Rs | 4.8 | 3.9 | 4.0 |  | 4.0 | 5.0 | 4.9 |
| Ho | 0.39 | 0.42 | 0.36 |  | 0.38 | 0.44 | 0.44 |
| Fis | **0.35** | 0.10 | **0.43** |  | 0.21 | **0.31** | 0.12 |
| **FUNF (3L)** |  |  |  |  |  |  |  |
| Rs | 8.4 | 6.8 | 7.0 |  | 7.3 | 7.0 | 6.0 |
| Ho | 0.81 | 0.70 | 0.74 |  | 0.71 | 0.74 | 0.72 |
| Fis | -0.04 | 0.09 | 0.02 |  | 0.03 | 0.02 | 0.01 |

Rs, allelic richness; Ho, observed heterozygosity; Fis, inbreeding coefficient (significant values after Bonferroni correction given in bold); sample sizes given in parentheses
